# Assessing socio-economic profile of U-Reporters: Towards establishing a pool for equity analysis of future crowdsourced surveys

**DOI:** 10.7189/jogh.11.09001

**Published:** 2021-03-01

**Authors:** James Powell, Christopher Brooks, Miku Watanabe, Lakshmi N Balaji

**Affiliations:** 1 Office of Innovation, UNICEF, Copenhagen, Denmark; 2 UNICEF country office in Bangladesh, Dhaka; 3 UNICEF Programme Division, New York, New York, USA

## Abstract

**Background:**

Crowdsourcing was recognized as having the potential to collect information rapidly, inexpensively and accurately. U-Report is a mobile empowerment platform that connects young people all over the world to information that will change their lives and influence decisions. Previous studies of U-Report’s effectiveness highlight strengths in the timeliness, low cost and high credibility for collecting and sending information, however they also highlight areas to improve on concerning data representation. EquityTool has developed a simpler approach to assess the wealth quintiles of respondents based on fewer questions derived from large household surveys such as Multiple Indicators Cluster Surveys (MICS) and Demographic and Health Surveys (DHS).

**Methods:**

The methodology of Equity Tool was adopted to assess the socio-economic profile of U-Reporters (ie, enrolled participants of U-Report) in Bangladesh. The RapidPro flow collected the survey responses and scored them against the DHS national wealth index using the EquityTool methodology. This helped placing each U-Reporter who completed all questions into the appropriate wealth quintile.

**Results:**

With 19% of the respondents completing all questions, the respondents fell into all 5 wealth quintiles, with 79% in the top-two quintiles and only 21% in the lower-three resulting in an Equity Index of 53/100 where 100 is completely in line with Bangladesh equity distribution and 1 is the least in line. An equitable random sample of 1828 U-Reporters from among the regular and frequent respondents was subsequently created for future surveys and the sample has an Equity Index of 98/100.

**Conclusions:**

U-Report in Bangladesh does reach the poorest quintiles while the initial recruitment skews to respondents towards better off families. It is possible to create an equitable random sub-sample of respondents from all five wealth quintiles and thus process information and data for future surveys. Moving forward, U-Reporters from the poorly represented quintiles may be incentivized to recruit peers to increase equity and representation. In times of COVID-19, U-Report in combination with the EquityTool has the potential to enhance the quality of crowdsourced data for statistical analysis.

Crowdsourcing of information was recognized as low-cost, rapid, with the potential to transcend many barriers and that it can be very promising in global health, particularly in resource poor settings with opportunities for real-time efficiencies and effectiveness on data collection and analysis [1]. Child Health and Nutrition Research Initiatives (CHNRI) method to generate and rank potential uses of crowdsourcing against a set of criteria identified Epidemic Response, Data generation, Problem solving as among the top 15 ideas. [2]. The current COVID-19 times are ideal for crowdsourcing applications to generate data. Reliable methods to collect statistically representative data from populations are still few or are not yet comparable to established surveys such as Demographic Health Surveys and Multiple Indicator Cluster Surveys.

U-Report [3]is a mobile-based crowd-sourcing communication platform to engage young people, designed to address issues that the population cares about. Through simple Short Message Service (SMS) messages and digital channels [including Facebook Messenger (Facebook Inc., Menlo Park, CA, USA), WhatsApp (WhatsApp Inc., Mountain View, CA, USA), Viber (Rakuten Viber, Luxembourg)], participants (also called as U-Reporters) can access the U-Report platform offering polls and useful information free of cost to the consumer or participant. U-Report is a user-centered social monitoring tool designed to trigger, promote and strengthen community-led development, citizen engagement and positive change. Data from U-Reporters has been found to be quick and useful with information often coming from the better literate and tech-savvy segments of the population, but not representative enough for any statistical analysis.

While individual messages exchanged on U-Report are confidential, aggregated data are transparent. Information received can be disaggregated by age, gender and country and by sub-national characteristics in real-time and is used to connect young people with their representatives, improve programmes for women, children and young people and draw attention to urgent issues with national and sub-national governments, the United Nations (UN), Non-governmental organizations (NGOs), Civil Society and country leaders who can see the information on the site to understand what the U-Reporter’s opinions or feedback.

It has been a challenge to make statistical sense of the data that is collected through U-Report via opt-in registration of participants, as opposed to selecting specific respondents to whom specific questions are asked, like in a traditional survey. Various attempts have been made to improve the statistical relevance and value of U-Report data. The most recent work relates to the work by Dhillon et al in 2017 [4] from the Lee Kuan Yew School of Public Policy for measuring perceptions using an analytical framework encompassing elements of (i) representation, (ii) timeliness, (iii) measurement and (iv) credibility. The authors note U-Report as being very high on timeliness while it needs to be improved on the representativeness. Further, the research also suggests that given the ready access of the analysis through the internet, the aggregated data was readily available for access and advocacy to key decision makers in the countries where it was tested. Representation and measurement were noted as challenges that need to be further explored and as areas that will require further attention.

So, the main question for improving the statistical value of the surveys conducted with the help of U-Reporters is – Is it possible to measure and therefore improve the ‘representativeness’ of a sample, without sacrificing either the timeliness or credibility already established (hitherto interpreted as a tradeoff) or the merits of the project to bring young people together and empower them to express themselves regardless of social stratification?

EquityTool was developed by Chakraborty et al in 2016 [5] as a simple and easy-to-use tool to measure relative wealth. The Tool uses a short survey and allows one to compare the wealth (measured through questions related to assets) of respondents to the national or urban population in over 30 countries. The questions that are normally used in a standard Demographic and Health Survey (DHS) and/or Multiple Indicators Cluster Survey (MICS) are ordered by statistical importance to the full wealth index, and added to the questionnaire in order, until a sufficient level of agreement is reached between the full wealth index and the simplified index that EquityTool has developed. The respondents are sorted into the same wealth quintiles by both indices at a level of agreement where kappa ≥0.75. A combination of U-Report and EquityTool in our assessment has the potential to increase the value and the representativeness of data generated by U-Report based surveys.

## METHODS

A large number of survey questions are required to construct the standard DHS wealth index, which is a challenge for any survey. This challenge is exacerbated in utilizing U-Report which is not a traditional survey tool and is best utilized when collecting fewer data points given that it works across the applications or channels that use smart phones or through use of short messaging service in a traditional mobile phone. The EquityTool survey was designed to simplify this research challenge and therefore has the potential to be combined with the U-Report methodology as it measures household wealth based on questions and items that are country specific and adjusted to the context and therefore likely to be more reliable. Once respondents are sorted into the five wealth quintiles, the variance from the population’s even quintile distribution can be measured. This enables an understanding of whether survey respondents are more, less, or equally wealthy compared to the national population.

**Figure 1:**
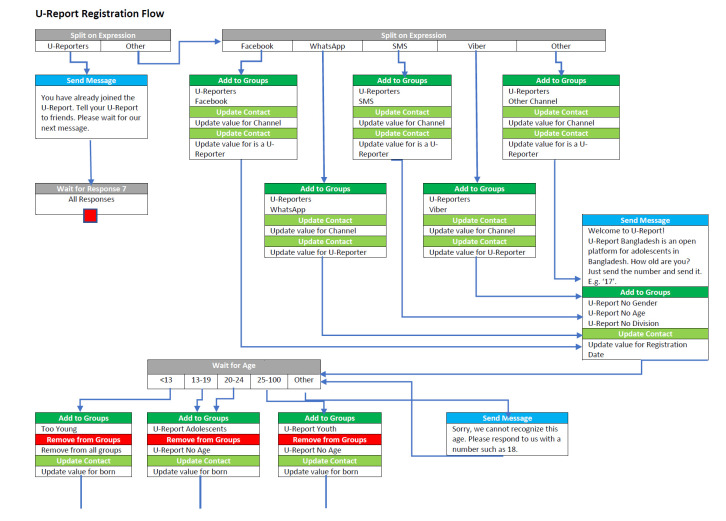
Example flow in RapidPro.

RapidPro (open source jointly owned by UNICEF and Nyaruka) is the software that powers U-Report. It is an open source messaging tool that uses a decision tree logic termed ”flows” that can segment and guide a participant through a series of messages and responses, collecting a data point at each “step” (**Figure 1**). These sequences of communication and data collection are visualized in flowcharts that are created by anyone operating the platform, in some cases youth groups with UNICEF support and oversight and in many cases vice versa. Responses are captured in “categories” designed and defined by the administrator.

**Figure 2:**
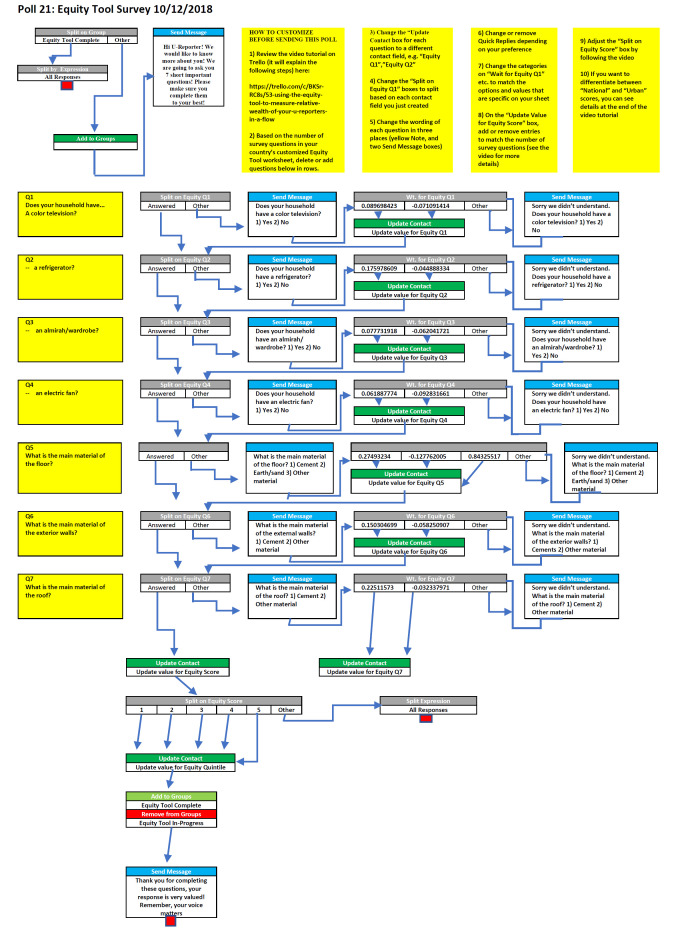
RapidPro flow of EquityTool survey with scoring mechanism.

Towards establishing a representative sub-sample of U-Reporters, it was decided to assess the socio-economic profile of U-Reporters in Bangladesh using the short pre-defined questionnaire developed by EquityTool for Bangladesh. Building on the preliminary RapidPro flow architecture developed and described by McFadden in 2018 [6], a RapidPro flow (**Figure 2**) that collected the survey responses, automatically scored them against the DHS national wealth index using the EquityTool methodology, and placed each U-Reporter into the appropriate wealth quintile.

### Poll Flow

The flow developed in RapidPro is based on an article posted by Kalee McFadden (The EquityTool and RapidPro, 2018). Users are taken progressively through a set of questions from the EquityTool survey for Bangladesh, and their responses are scored and combined to produce a wealth index comparable to the Demographic Health Survey for Bangladesh, which was taken as a reference for each of the respondents in U-Report. That wealth index is then scored against the wealth quintile bands for Bangladesh and the user is automatically placed into one of the five quintiles.

The newly designed flow makes several material improvements over the original:

- The score for each question is saved in an individual contact field, which allows for:
  - resuming of the survey later,
  - potential rescoring of respondents from national to urban quintiles;
- The overall wealth index is stored in a contact field which allows for adjusting respondents into different quintiles in the event a new EquityTool scoring is released based on a new survey, as long as the questions are still the same;
- The quintile score is stored in a contact field, which allows for:
  - analysis of future polls by respondent quintile,
  - potential long-term tracking of users’ wealth via new waves of the survey,
  - easy access and review by U-Report project staff;
- The flow is designed to be resumable, meaning that when a respondent enters the flow a second time they will continue to be presented with survey questions:
  - future versions of the flow will include a marker for the respondent’s start date in the survey, to ensure that it is completed within an acceptable timeframe.

**Figure 3:**
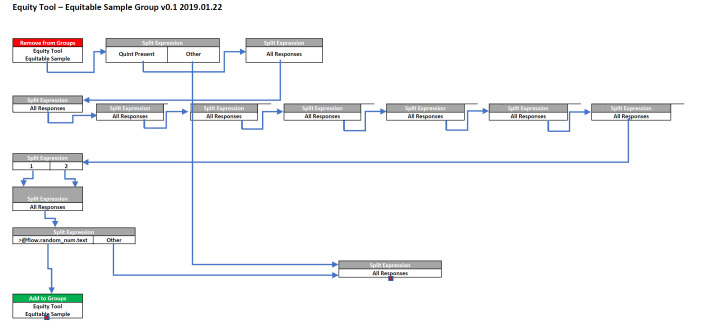
Equitable sample group creation flow.

With the knowledge of each respondents’ wealth quintile assigned to their profiles, we can build a sample of U-Reporters representing equitable wealth distribution for each country participating. This was accomplished by a newly developed RapidPro flow (**Figure 3**). Importantly, RapidPro is a multi-tenant system whereby each U-Report country has their own workspace based on the core RapidPro software, as such a flow created in one can be easily imported and adopted in another, creating ideal conditions for portability and scale up. Therefore, each flow can easily be deployed by U-Report project managers responsible for their country after they have implemented their own EquityTool surveys.

The first pilot of EquityTool was carried out in U-Report Bangladesh in December 2018. It was decided to pilot this initiative in Bangladesh as U-Report platform has a mix of channels, viz. SMS, Facebook Messenger, Viber and WhatsApp and the platform was in its early phase of the life cycle having launched U-Report in the country just a few months prior in July 2018. U-report Bangladesh scale up is characterized by focusing on registering opt-in U-Reporters via digital channels rather than SMS in the early phases of scale up, then SMS started later once partnership agreements had been finalised. Based on the hypothesis that people in Bangladesh with internet access are more likely to be in wealthier quintiles, it was decided this would be an ideal candidate for piloting and testing the theory of quintile categorization and redistribution into equitable samples.

### Data Analysis process

#### 1. Exporting data from RapidPro

For simplicity, data was exported using the RapidPro web interface.

1. Contact data was exported in full, with the addition of groups for Equity Tool Started, Equity Tool Completed, and the group for each RapidPro channel, eg, “Viber”, “Facebook”, etc.

2. Flow results were exported in full, with the addition of:

- Groups for Equity Tool Started, Equity Tool Completed, and the group for each RapidPro channel, eg, “Viber”, “Facebook”, etc.;
- Fields for “Born” (i.e., the year participants were born), “Division”, and “Gender”.

#### 2. Normalization using configuration spreadsheet

Each U-Report project uses slightly different formats for its data, so to generalize the data ingestion as much as possible, we developed a configuration file that could be used for cleaning and re-coding the raw data. For Bangladesh, the structure was as noted in **Table 1**.

**Table 1:** Configuration spreadsheet for Bangladesh

Field	Data	Type	Standardized	Name_in_Rapidpro
Gender	Structure	Field	gender	Gender
Age	Structure	Field	age	
Born	Structure	Field	born	Born
Language	Structure		language	Language
Location Fields (list)	Structure	Field		Division
Channel	Structure	Field	channel	Channel
Equity Tool Status	Structure	Field	et_status	
Equity Tool Start Date	Structure	Field	et_start_date	
Equity Tool Complete Date	Structure	Field	et_complete_date	
Equity Tool Score	Structure	Field	et_score	Equity Score
Equity Tool Quintile	Structure	Field	et_quintile	Equity Quintile
Equity Tool Questions (list)	Structure	Field		Equity Q1, Equity Q2, Equity Q3, Equity Q4, Equity Q5, Equity Q6, Equity Q7
Other Fields to Keep (optional – list)	Structure	Field		Upazila, Union, Club Name, Partner
Age Target Min	Project		13	
Age Target Max	Project		24	
Age Bands	Project		13,18,24,30	

The EquityTool survey questions were administered to 56 800 U-Reporters via the communication channels connected to U-Report Bangladesh: Facebook (77%), SMS (20%), WhatsApp (2%), Other (1%), Viber (<1%). Participation in the survey was voluntary and free of cost to the respondents, and a response was not required for users’ continued participation in U-Report. Just under three days (71.7 hours) after the survey was sent, the participants were removed from the flow, preventing recording of late responses. This essentially “closed” the flow.

Per the EquityTool methodology for Bangladesh, only those who answered all 7 questions in the survey are included in the quintile analysis. Based on the Bangladesh DHS, the following seven questions were selected by EquityTool to determine the respondents’ wealth quintile:

1. Does your household have a television? Yes/No;
2. Does your household have a refrigerator? Yes/No;
3. Does your household have an almirah/ wardrobe? Yes/ No;
4. Does your household have an electric fan? Yes/No;
5. What is the main material of the floor? Cement/ Earth or Sand/ Other material;
6. What is the main material of the exterior walls? Cement/Other material;
7. What is the main material of the roof? Cement/Other material;

The users’ responses to each question were scored and summed into a wealth index, then scaled against upper and lower boundaries for each of the wealth quintiles. This process was carried out in RapidPro through the following automated system:

- A U-Reporter responds to each question, and these responses are categorized in RapidPro into the scores based on the classification from the EquityTool;
- Once all questions are answered, and all scores are collected, the U-Reporters are sent through a series of operations in the flow that use this score data to identify the appropriate wealth quintile:
  - First all scores are summed to create the wealth index for the user,
  - Then the wealth index is compared to a series of upper and lower boundaries for each wealth quintile. For example, if the boundaries for the middle wealth quintile are 0.03 (upper) and -0.03 (lower), and the user’s wealth index is 0.00, that user will be placed in middle wealth quintile;
- Finally, the information on wealth quintile so determined is saved into a contact field for the user which means the administrator of the system will know going forward which wealth quintile the user belongs to – subsequent polls or communications can now be targeted by this information or analysis of future surveys disaggregated using this information.

Excel spreadsheets of RapidPro contact information and flow results for the EquityTool flow were are then exported for analysis and visualization in RapidPro.

## RESULTS

**Figure 4:**
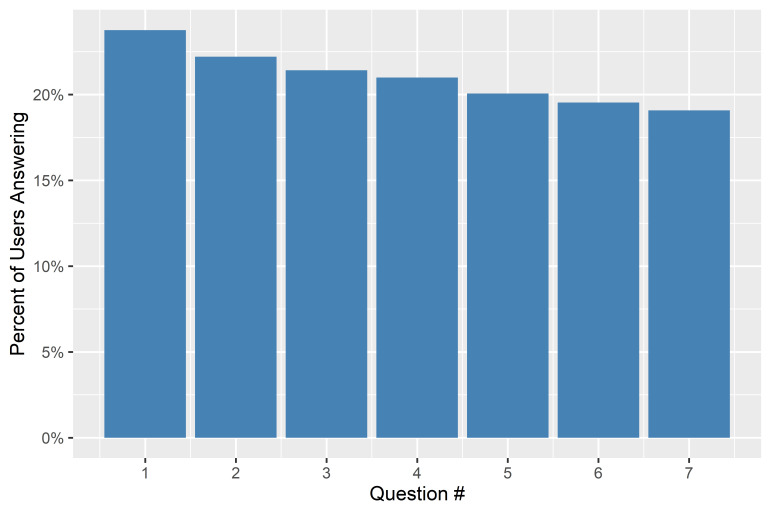
Survey completion.

Of the 56 800 users that received the survey, 10 837 completed it fully (19%), with the majority of U-Reporters who started the poll answering all 7 questions (**Figure 4**).

23.8% of users began the poll, and 80.3% of those who began the survey completed it.

### Survey results

**Figure 5:**
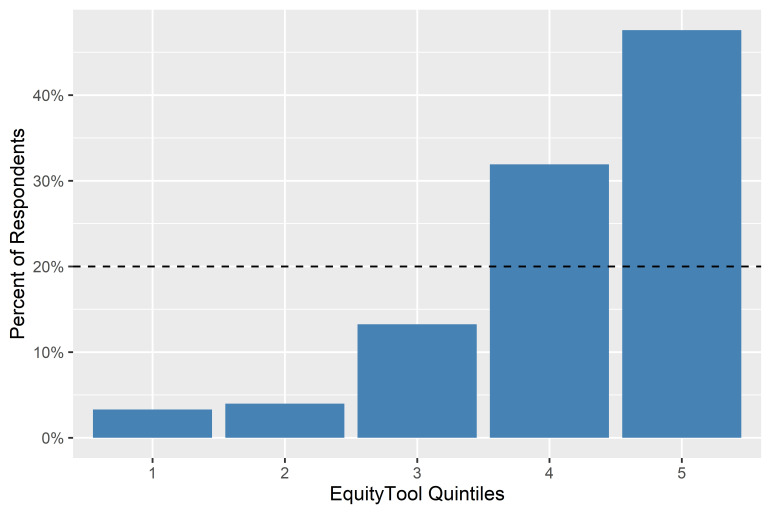
Quintile distribution for all EquityTool respondents.

The EquityTool poll respondents were shown to fall into all 5 wealth quintiles with 79% in the top-two quintiles and 21% in the lower-three (precision of +/− 1%) suggesting that a majority of the U-Reporters who recruit themselves into the network primarily through digital channels in Bangladesh are unsurprisingly more affluent than the distribution one would expect from a random sample as demonstrated in **Figure 5**.

#### 1. Survey dropoff analysis

The percentage of respondents that successfully answered each survey question were tabulated and visualized.

#### 2. Quintile distribution comparison

The distribution of wealth quintiles for the sample was visualized compared to the population distribution of even quintiles, and it was determined that the respondents from U-Report Bangladesh were more skewed toward the higher wealth quintiles than the Bangladesh population. A Gini coefficient analysis was performed on the quintile results, providing a coefficient of 0.47. For the purposes of this paper, that coefficient was converted into an “Equity Index” via the formula (1-.47) × 100 = 53.

#### 3. By-channel quintile distribution comparison

The respondent sample was then broken down by communications channels. Among channels with over 384 individual respondents, corresponding to ±5% accuracy according to EquityTool’s sampling instructions, [7], wealth quintile distribution was found to be most equitable among SMS users and least equitable among WhatsApp users.

#### 4. Equitable sub-sample quintile distribution

After the equitable sub-sample was created, a contact export was performed in RapidPro in order to retrieve the list of Contact Unique User Identifications (UUIDs) present in the sub-sample. The full sample of respondents was then filtered by those Contact UUIDs to produce a comparison between Equity Index of the original sample and the sub-sample.

**Figure 6:**
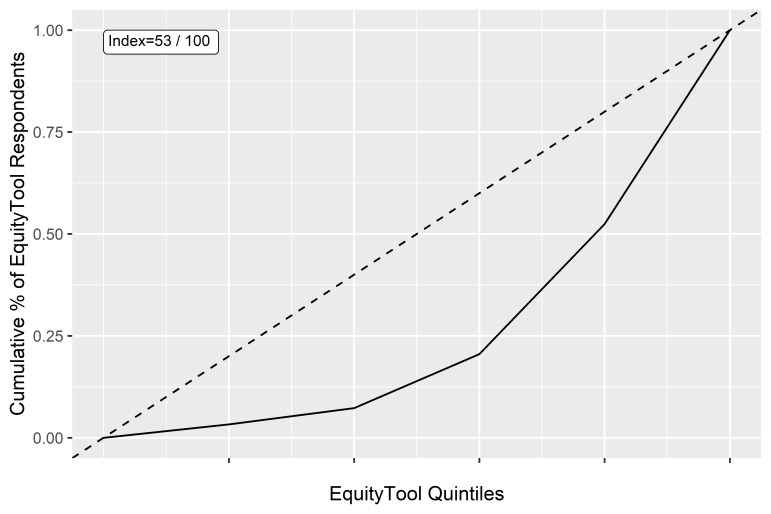
Lorenz curve and equity index for all equity tool respondents.

This variance from equal quintile distribution has an Equity Index of 53, on a 0-100 scale derived from the Gini index where 100 is wealth distribution perfectly matching the population (**Figure 6**).

**Figure 7:**
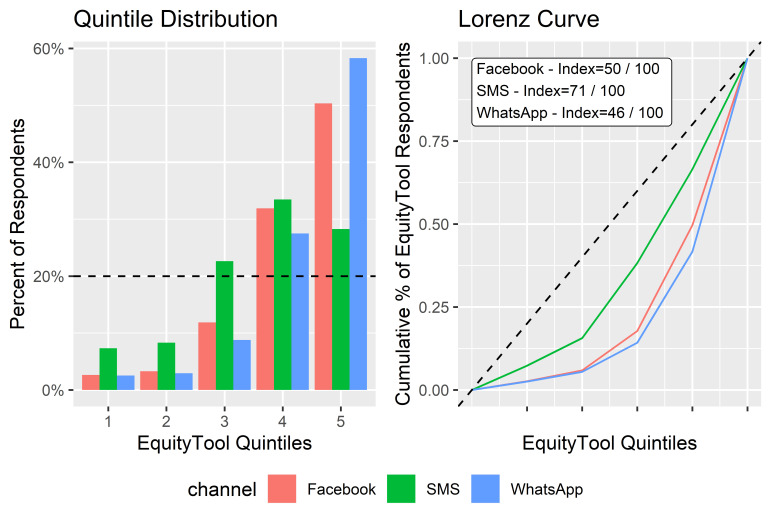
Quintile distribution, Lorenz curve, and equity index by communication channel.

Among the various channels used by U-Report Bangladesh, SMS was shown to be the most equally distributed among the quintiles, WhatsApp the least as shown below in **Figure 7**.

Respondents using Viber, and “Other” channels were not compared as the numbers were too small. As suggested by the EquityTool’s Excel tool for sampling design, a sample of 384 respondents was required to achieve an accuracy of +/− 5%.

### Post-adjustment and equitable sample creation

With 10 837 U-Reporters successfully completing all questions of the EquityTool survey, it was possible to randomly re-sample a group of U-Reporters that represent an equitable cross-section of the country’s population by wealth.

Thus, we created a post-adjusted sub-sample of size 1828, which can be used in the future for surveys that require an equitable distribution across all wealth quintiles. The RapidPro flow shown in **Figure 3** was used to select such a sub-sample:

- The total number of respondents in each wealth quintile is manually input into a “Split By Expression” box;
- Based on the size of the smallest quintile, the flow determines what percentage of each larger quintile should be randomly sampled into the equitable sample in order to achieve equal size of each wealth quintile,
  - For example, if the smallest quintile has 100 respondents, and the largest quintile has 1000 respondents, 10% of the largest quintile should be included at random in the equitable sample;
- The flow then assigns a random number between 0 and 1 to the user;
- If the percentage, converted to decimal, is larger than the user’s randomly assigned number, then that user will be added to the equitable sample;
- There will be a small variation in the total number of respondents assigned to each quintile due to the random nature of this selection.

**Figure 8:**
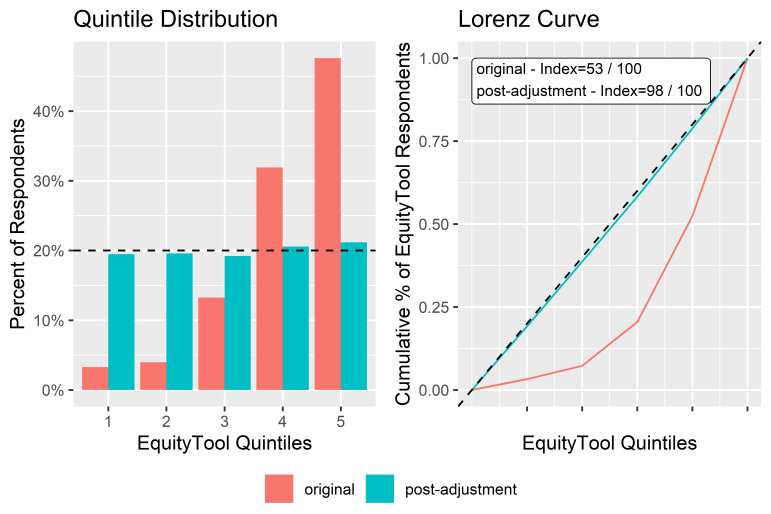
Lorenz curve, and equity index for all respondents vs post-adjustment equitable sample.

The result of this process shown in **Figure 8** is a sample composed of the entire sample from the smallest quintile, and a nearly-equal number of respondents from each of the other quintiles. The resulting equitable sample has an Equity Index of 98.

## DISCUSSION

### Peer-referral recruitment initiatives for inadequately represented quintiles among the U-Reporters

With knowledge of which U-Reporters fall into the most marginalized and under-represented quintiles, we recommend pursuing a peer-referral recruitment initiative targeted to increasing the number of respondents belonging to the least well represented quintiles. In this case, the bottom-three quintiles had fewer number of respondents when compared with those in the better off 40% (2 quintiles). RapidPro flows have already been designed and tested to execute and expedite this type of recruitment drive. There is an implicit assumption that those belonging to the same socio-economic strata will generally motivate and enroll those from among similar socio-economic profile, which will require further research and verification.

### Diversification of recruitment among channels

Users of the SMS channel were more equitably distributed among the wealth quintiles than users of digital channels. We offer the following explanations for such a distribution:

- Access to digital channels (eg, Facebook Messenger, WhatsApp, Viber) requires internet access,
  - Access may be infrequent or unavailable especially for those belonging to the families who are poor or those communities who are marginalized,
  - While data usage by digital messaging channels may be minimal, a data package purchase may still be required;
- Not all feature phones support digital messaging channels;
- Recruitment for digital channels in Bangladesh to date has relied heavily on Facebook advertisements, which skews toward heavier users of Facebook and people with easier/more consistent internet access.

Conversely, SMS channel users’ wealth may be more equitable because:

- There is no internet access requirement for SMS users;
- SMS is available on most feature phones;
- Recruitment relied heavily on push messaging, which doesn’t pre-suppose usage of Facebook or another advertisement-supported platform.

With this in mind, we recommend pursuing a diverse channel and recruitment approach. Both SMS and digital channels are important - the former for reaching individuals and families who belong to the marginalized segments of the society, and the latter for engaging users that primarily use digital channels and may not engage via SMS.

## CONCLUSION

U-Report in Bangladesh has a better understanding of the challenges related to the distribution of users across different socio-economic strata. It is possible to choose from among those who are regular users and respondents of U-Report, a sub-sample that is evenly distributed across the five wealth quintiles and thus improves the ‘representativeness’ of future surveys. Such a strategy increases the value of the data generated by the community, as well as its potential for use and application to programme planning, accountability mechanisms and monitoring.

Despite a tendency to skew self-recruitment of users of U-Report from among the wealthier quintiles, the U-Report in Bangladesh can indeed reach out and make special efforts to promote individuals from the under-represented quintiles and thus improve the distribution of respondents. It has been possible to establish a sub-sample of respondents distributed equally among all five quintiles in Bangladesh and they can be polled in future to ensure representative pool to better understand U-Reporter’s voices and data when required. This can be used without negatively impacting on the timeliness of the data as it has been built within the system. Finally, UNICEF Bangladesh and their partners can use the findings to adjust their recruitment techniques to continuously improve representation in the future.

Moving forward, as the enrolment of U-Reporters in Bangladesh improves, similar strategies can be adopted to further sharpen ‘representativeness’ of polling and survey data not only for national surveys, but also for sub-national surveys by divisions to provide critical programme and monitoring information for sharpening the universality of programmes and thus help the country pursue Sustainable Development Goals across all social development areas. It is hoped that this approach will considerably enhance the rigour and statistical analysis for crowd-sourced data coming from sources such as U-Report.
